# Diffuse lesions secondary to sarcoidosis mimicking widespread metastatic breast cancer: A case report

**DOI:** 10.1002/ccr3.3561

**Published:** 2020-12-01

**Authors:** Heidi Vieira, Beth K. Neilsen, Richard Sleightholm, Jordan Hankins, Alison Freifeld, Gerald Moore, Andrew Wahl, Michael J. Baine

**Affiliations:** ^1^ Department of Radiation Oncology University of Nebraska Medical Center Omaha NE USA; ^2^ Department of Radiology University of Nebraska Medical Center Omaha NE USA; ^3^ Department of Internal Medicine University of Nebraska Medical Center Omaha NE USA

**Keywords:** infectious diseases, oncology

## Abstract

This case of sarcoidosis mimicking metastatic breast cancer serves as a reminder of the need to consider differential diagnoses even when the clinical scenario and imaging findings are highly suggestive of metastases.

## Graphical abstract

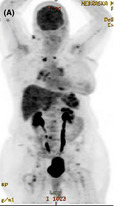

## 1 INTRODUCTION

A 71‐year‐old woman with a history of breast cancer presented with back pain and diffuse PET‐avid lesions consistent with diffuse metastatic disease but was found to represent biopsy‐proven sarcoidosis. This highlights the need for a broad differential even when the clinical scenario and imaging findings are highly suggestive of metastases.

Breast cancer frequently metastasizes to lymphatics, bone, lung, liver, and brain, with bone being the most common hematogenous route and representing more than 70% of distant metastases.^1^ Risk factors for bone metastasis include advanced clinical stage, lymph node metastases, negative progesterone receptor status, and tumor subtype.^2^ On CT imaging, metastatic bone lesions can appear as either osteolytic or osteoblastic, or they can have a mix of osteolytic and osteoblastic features; they are most commonly located in the vertebrae and pelvis owing to the rich vascularization of these areas.^3^ There are, however, several other conditions that can mimic the imaging findings of metastatic cancer and should be ruled out prior to ascribing the changes seen on imaging to metastatic spread.

One such condition is sarcoidosis, an inflammatory condition with highly variable clinical presentation and a hallmark pathological finding of non‐caseating epithelioid granulomatous inflammation. Sarcoid granulomas most often affect the lungs and lymph nodes in >90% of cases, but can be present in almost any tissue in the body.^4^ Bone lesions are present in an estimated 3%‐13% of cases, most commonly in the small bones of the hands and feet; vertebral involvement is rare but has been reported.^5^ Vertebral lesions have variable appearance on CT, with existing reports describing lytic, mixed lytic with sclerotic features, and sclerotic appearance.^6^, ^7^ Much like metastases and some infectious granulomas, sarcoid granulomas display increased avidity for fluorodeoxyglucose (FDG) during PET/CT imaging.^8^

Herein, we present a case of a 71‐year‐old female with past medical history significant for breast cancer who was thought to have widely metastatic disease based on clinical picture and imagining findings, but was instead found to have an inflammatory cause for her widespread hypermetabolic bone and soft tissue lesions on biopsy.

## 2 CASE PRESENTATION

A 71‐year‐old Caucasian female presented with a chief complaint of acutely worsened lower back pain. The pain had come on over the previous two months and was localized over the upper lumbar spine. She reported an increase in intensity of the pain when lying flat. The patient had a longstanding history of chronic back pain, and two previous surgeries for adult tethered cord syndrome, but reported this pain to be significantly worse than what she had experienced before.

Notably, her past medical history included a diagnosis 13 months prior of infiltrating ductal adenocarcinoma of the breast, which had been identified on routine screening mammogram. The tumor was determined on core needle biopsy to be ER (90%), PR (95%), and Her2 (3+) positive, with a Ki67 of 13%. She had undergone a left breast lumpectomy and sentinel lymph node biopsy with negative surgical margins. Final pathology from lumpectomy demonstrated the tumor was grade 2 and 1.2 cm in greatest dimension. Focal DCIS was present with high nuclear grade. The sample was without lymphovascular invasion. Pathology showed one of six sentinel nodes was positive for macrometastatic disease with associated extracapsular extension. Final pathological staging after lumpectomy and sentinel lymph node biopsy was pT1N1aMx.

After lumpectomy, the patient had been treated with adjuvant chemotherapy followed by radiation therapy and hormonal therapy. Her chemotherapy regimen consisted of dose dense paclitaxel, cyclophosphamide, and trastuzumab for a total of six cycles, which was complicated by neutropenic fever following the first dose as well as a persistently infected wound of the toe prompting a dose reduction of paclitaxel and cyclophosphamide for the remaining five cycles, with plans to continue trastuzumab therapy for one year. Her radiation treatment was initiated five weeks following completion of her cyclophosphamide and paclitaxel treatment. Radiation treatment was delivered to the whole breast with high tangents using a hypofractionated course to a total dose of 4256 cGy delivered over 16 fractions followed by a 1000 cGy boost to the tumor bed delivered over five fractions. Given the ER‐positive status of her tumor, the patient was also started on anastrozole at the conclusion of her radiation therapy. Ten months after initial diagnosis and three months after the completion of radiation therapy, a diagnostic mammogram showed no evidence of disease in either breast. The patient was continued on trastuzumab and anastrozole.

Her presentation with acutely worsened back pain occurred one week after completion of the one‐year of adjuvant trastuzumab therapy, and approximately 13 months after breast cancer diagnosis. A review of systems was negative for constitutional symptoms. Physical examination at the time of presentation showed tenderness to palpation midline in the upper lumbar spine, with limited range of motion bidirectionally due to pain. Neurological examination, including assessment of strength and gait, was normal. No laboratories were drawn at this time.

**Figure 1:**
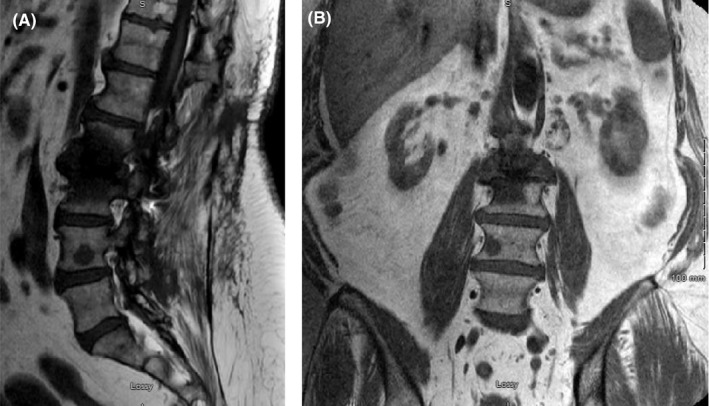
Representative sagittal (A) and coronal (B) MRI sections demonstrating lesions at L2/3 and L4

**Figure 2:**
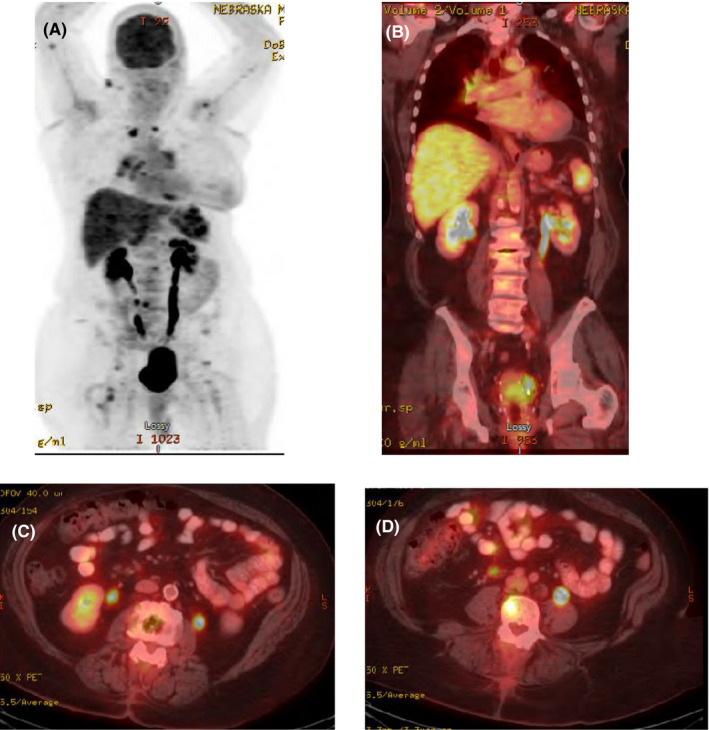
Representative images from PET/CT scan. (A) PET/CT composite. (B) Coronal section on PET/CT. (C) Axial section of L2/3 lesion. (D) Axial section of L4 lesion

An MRI of the spine was ordered, which showed an abnormal signal and enhancement at L2, L4, and S1 in addition to iliac and sacral lesions consistent with metastatic disease (Figure 1). A follow‐up PET scan demonstrated multifocal hypermetabolic lesions in the mediastinum, hila, spleen, liver, abdominal and inguinal lymph nodes, as well as in multiple bones (SUV_max_(range): 3‐5.75) suggestive of widespread and distant metastatic involvement (Figure 2). A biopsy of a liver lesion was planned for confirmation of metastatic disease but results from this failed to confirm the diagnosis of metastasis and showed no signs of malignancy, instead unexpectedly showing granulomatous hepatitis with multiple non‐caseating epithelioid granulomas.

Due to these non‐confirmatory findings on biopsy, the case was reviewed by a multidisciplinary tumor board comprising diagnostic radiology, pathology, oncology, and radiation oncology. The consensus from this session was that, despite the nondiagnostic biopsy results, the patient's imaging findings, clinical findings, and history of breast cancer were most consistent with metastatic disease and palliative radiation therapy with repeat biopsy of a separate site was recommended.

Two weeks later, the patient underwent a CT‐guided biopsy of the left iliac and right side of the L4 vertebral body. These biopsies again failed to demonstrate any evidence of malignancy but instead consistently demonstrated a non‐necrotizing granulomatous pattern of disease. Therefore, metastatic disease was no longer considered the likely diagnosis due to the biopsies of multiple sites failing to demonstrate any evidence of metastatic disease.

A comprehensive workup was performed to evaluate for a possible infectious source for the lesions. A quantiferon test as well as urine and serum Ag/Ab tests for histoplasma, blastomycosis, coccidiomycosis, and treponema was completed with all tests coming back negative. PCR testing of the liver was also negative for acid‐fast bacilli and fungi. On further evaluation, it was noted that one year prior to her breast cancer diagnosis, the patient had complained of a persistent cough with evaluation demonstrating imaging evidence of possible sarcoidosis. She did not receive treatment for sarcoidosis at the time, and instead was instructed to return if her cough worsened. As a result of this additional information and her recent biopsy results demonstrating non‐necrotizing granulomas, the patient was referred to a rheumatologist, and her imaging findings were evaluated for their potential to represent widespread sarcoidosis. However, she still refrained from initiating steroid treatment for sarcoidosis as her back pain was thought to be more consistent with worsening osteoarthritis after consultation with a rheumatologist. A repeat MRI of the lumbar spine four months later demonstrated that the PET‐avid bone and soft tissue lesions were stable and comparable in size with what was seen in the previous MRI, providing further reassurance against a metastatic etiology for these lesions.

## 3 DISCUSSION

This case report should serve as a cautionary tale describing alternative potential causes of imaging findings consistent with malignancy. Multiple groups have previously described additional cases or highlighted other causes of PET‐avid lesions that mimic malignancy on PET/CT scan that were largely observed during initial cancer staging or incidental findings on imaging. In this case, the patient presented with numerous lesions more than a year after her initial breast cancer diagnosis mimicking a more recurrent/metastatic clinical picture. Our patient presented with back pain, a common presenting symptom in patients with bone metastasis, and was then found to have multiple bone lesions on spine MRI and additional soft tissue involvement on subsequent PET imaging. Based on her past medical history, clinical picture, and corroborating imaging findings, this patient was believed to have metastatic disease for several weeks while awaiting biopsy results that revealed a nonmalignant etiology.

Known nonmalignant causes of FDG‐avid lesions include anything that induces inflammation or increased glucose uptake in tissue, which are commonly of infectious or autoimmune origin. Other groups have reported false positive PET scan findings can be caused by infectious diseases (including mycobacterial, fungal, or bacterial), sarcoidosis, trauma, and post‐operative surgical conditions in the absence of malignancy.^9^, ^10^, ^11^, ^12^, ^13^, ^14^, ^15^, ^16^, ^17^, ^18^, ^19^, ^20^ In fact, a concurrent diagnosis of sarcoidosis has been described in a case report when additional lesions were identified during initial cancer staging workup, but the additional lesions were subsequently determined to be nonmalignant.^21^ Other alternative nonmalignant etiologies of bone lesions include fibrous dysplasia of bone, osteonecrosis, osteitis fibrosa cystica, and Paget's disease of bone, among others, can present with PET‐avid lesions on imaging and characteristic laboratory findings including hypercalcemia.^10^, ^13^, ^14^, ^15^, ^22^ Infectious etiologies also need to be ruled out when PET‐avid bone lesions are identified as disseminated tuberculosis and multifocal osteomyelitis have both been reported to be mistaken for metastases on PET scan.^12^, ^16^, ^18^

Multiple cutoff values have been proposed to distinguish between benign and malignant origins for increased FDG‐avidity. One group proposed an SUV cutoff of 3 for bone metastases with sensitivities ranging from 95.2% to 99.6% and specificity ranging from 75% to 100% for various primary malignancies including breast.^23^ Another group evaluating rib metastases found the max SUV was higher in malignancy (3.0 ± 1.8) than in benign causes (2.5 ± 1.1), but had significant overlap and recommended a cutoff of 2.4.^24^ Finally, in an evaluation for axillary metastasis as part of the initial workup for newly diagnosed breast cancer, a max SUV cutoff of 2.3 had a sensitivity of 60% and a specificity of 100%. However, no single cutoff is reliable in all circumstances and the range for benign vs. malignant causes overlap. For example, in our patient the SUV_max_ was 5.75 and 4.91 in two of her bone lesions with numerous areas of soft tissue and lymph nodes having an SUV_max_ above 3.

While more qualitative metrics, the pattern of disease and other specific imaging findings can be used to try to distinguish between possible etiologies. For example, while the radiographic findings in sarcoidosis are highly variable, it tends to favor a symmetric and central distribution. Another radiographic finding that is commonly attributed to metastatic disease is increasing lymph node size. However, increasing size of lymph nodes on imaging can also have a range of non‐metastatic causes including sarcoidosis, fibrosing mediastinitis, or even a second, primary lymphoma.^11^, ^17^, ^25^

In this case, the patient's clinical presentation and history were consistent with metastatic disease, but her back pain and hypermetabolic lesions were ultimately determined to be nonmalignant in nature representing likely musculoskeletal and sarcoid‐related findings, respectively. Thus, the differential diagnoses in a patient with newly identified lesions on PET/CT should be evaluated according to the clinical scenario as well as specific imaging findings (level of SUV_max_, CT or MRI findings). However, without confirmation from tissue biopsy there will always be at least a small amount of uncertainty. Therefore, lymph node and other image‐identified lesions should be biopsied whenever possible to confirm suspected metastases. Unfortunately, such a biopsy may not always be feasible due to complex anatomic location, patient history, or other complicating factors, could delay treatment, and therefore may be omitted in the setting of a clear clinical picture, patient history, and/or inability to obtain tissue biopsy.

## 4 CONCLUSIONS

In this case, the consistency of the patient's clinical and imaging findings with metastatic disease and the much higher likelihood of metastatic disease vs. other causes in this clinical scenario supported the use of empiric therapy. Regardless, the possibility of another etiology causing similar findings on imaging should be considered and ruled out if at all possible prior to treatment. Empiric radiation therapy is commonly used in the setting of previously biopsy‐proven malignant disease for the purpose of pain palliation or for the prevention of impending anatomic damage (ie, spinal cord compression). However, the likelihood of metastatic disease and urgency of the clinical scenario should drive decision‐making in these situations. Ideally, timely imaging in conjunction with pathologic evidence of malignant disease should be obtained prior to any treatment if at all possible. Unfortunately, however, this is not always possible or routinely practiced in the setting of probable metastatic disease inducing significant pain or impending spinal cord compression.

Overall, this case highlights the need to consider a wide differential of potential diagnoses even when the clinical scenario and imaging findings are highly suggestive of metastases. In addition, prompt biopsy confirmation of metastatic disease provides significant value through confirmation of the diagnosis even when the clinical and imaging data are suggestive thereby ensuring appropriate treatment.

## CONFLICT OF INTEREST

The authors declare no conflicts of interest.

## AUTHOR CONTRIBUTIONS

HV, BN, and RS: reviewed patient records, drafted the case report, and revised the final manuscript. JH, AF, GM, AW, and MB: participated in direct patient care and provided the patient background and presentation. All authors: revised the final manuscript and gave final approval for publication.
